# An Earthquake Emergency Web Data Cleaning and Classification Method Based on Word Frequency and Position Weighting

**DOI:** 10.1155/2022/6555392

**Published:** 2022-09-10

**Authors:** Shuai Liu, Meng Huang, Chenxi Li, Wenchao Lv, Zhonghao Wang

**Affiliations:** Institute of Disaster Prevention, Sanhe, Hebei, China

## Abstract

The speed of earthquake emergency web document data cleaning is one of the key factors affecting emergency rescue decision-making. Data classification is the core process of data cleaning, and the efficiency of data classification determines the speed of data cleaning. This article is based on earthquake emergency Web document data and HTML structural features, combined with TF-IDF Algorithm and information calculation model, improves the word frequency factor and location factor parameters, and proposes the weighted frequency algorithm P-TF-IDF for earthquake emergency Web documents. To filter out less frequent words and optimize the FastText model, N-gram Feature word vectors effectively improve the efficiency of Web document data classification; for text classification data, use missing data recognition rules, data classification rules, and data repair rules to design an artificial intelligence-based earthquake emergency network information data cleaning framework to detect invalid data sets value, complete data comparison and redundancy judgment, clean up data conflicts and data errors, and generate a complete data set without duplication. The data cleaning framework not only completes the fusion of earthquake emergency network information but also provides a data foundation for the visualization of earthquake emergency data.

## 1. Introduction

Earthquake emergency work is a paramilitary operation, and the key to success or failure lies in whether scientific and reasonable decisions can be made and put into action in the shortest possible time. Scientific and reasonable decision-making requires comprehensive, accurate, and timely disaster information support. Accurate disaster information is also a key factor in guiding public opinion involving earthquakes. The integration of artificial intelligence technology and earthquake emergency network information has become possible to quickly obtain massive network disaster information after the earthquake, and the basis for emergency response and timely rescue decision support is becoming more and more comprehensive and rich.

At present, the construction of emergency response information system is lagging behind, and emergency rescuers have a heavy workload for information processing of sudden earthquake events, which seriously hinders the efficiency of emergency response. Especially for the massive amount of earthquake disaster information that cannot be collected and processed in a timely and effective manner. Taking network resources as an example, there is special network information of the earthquake industry, news media websites, microblogs, forums, etc., and a large amount of useful disaster information is mixed in various forms. Among the massive data, in addition, the information often contains a large amount of “dirty data” (such as information duplication, error, incompleteness, inconsistency of information, and logic errors). Therefore, how to use artificial intelligence technology to quickly detect the “dirty data” in the disaster data, and effectively clean and organize them is important, so that the earthquake emergency scientific research personnel can carry out the next analysis and research, which is one of the key problems to be solved in the earthquake emergency work at the present stage, especially after the acquisition of large earthquake disaster.

Organize data before data cleaning is particularly important, among which data classification is one of the important means of organize data. Recently, Dr. Mario et al. proposed a completely innovative fuzzy classifier based on fuzzy similarity calculation, and implemented a low-cost analysis system including probe on hardware, which effectively improves the efficiency of airline maintenance of aircraft safety standards [1]. The research on data cleaning first appeared in the United States, starting from the correction of the national social security number error, mainly by introducing data mining methods, using backtracking ideas, analyzing the characteristics of data sources, and analyzing data according to the corresponding business rules. Analyze and inspect each link, summarize the corresponding data cleaning rules and strategies, and extract data cleaning strategies, algorithms, and frameworks that meet the actual needs based on the existing cleaning algorithms and models, and finally on the data set. Apply these algorithms to restore the data to the data that can be used by the user. For example, the clustering method is used to detect abnormal records, the model method finds records that do not conform to the existing pattern, and then the association rule method is used to find abnormal data to detect and eliminate abnormal and approximate duplicate records. At the same time, with the development of Internet information, the research on data cleaning technology has been promoted. For example, in terms of network data cleaning, it is mainly divided into two categories: web page level data cleaning and page internal element level data cleaning. The former is represented by the PageRank algorithm proposed by Google and the HITS algorithm of the Clever system of IBM. The idea of the latter is mainly reflected in the VIPS algorithm, which is one of the core technologies of MSN Search engine [2–5].

In 2006, He Qing, an associate researcher of the Intelligent Information Processing Laboratory of the Institute of Computing Technology, Chinese Academy of Sciences, established a noise removal strategy for web page data cleaning based on the interference of web page noise data on web mining. This strategy established a noise discrimination model based on HTML markup information and the noise discrimination model based on data redundancy and the noise discrimination model based on the information entropy of the marked text perform noise removal. In 2010, Xiangfeng Meng, Air Force Radar Academy, proposed a similar repeated record detection method based on genetic neural network in order to effectively solve the detection problem of similar duplicate records in the field of web data cleaning. This method calculates the similarity between the corresponding fields of two records, builds a neural network-based detection model, uses genetic algorithm to optimize the weight of the network model, and uses genetic neural network to combine similarities on multiple fields to detect similar duplicate records. In 2017, Associate Researcher Jiangong Song of Beijing University of Aeronautics and Astronautics proposed an Internet information processing method for earthquake emergency response, and designed a set of web cleaning algorithm EESRC, which defines the priority of data attributes and calculates the corresponding weight; the data is weighted. The highest attribute fields are clustered to form several small data sets. The LP algorithm is used to cluster and merge them into a set, and then all the classes are individually matched for multiple times. For repeated records in different groups, choose to include high relevance to topic words. The attribute value records select the most recent web page update time and keep them, and delete the rest to generate a complete data set without duplication [6–12].

Earthquake emergency network information provides strong data support for the acquisition and analysis of earthquake disasters. However, there are few researches on the cleaning of “dirty data” related to earthquake emergency networks. Although some experts have conducted some research on data cleaning and analysis in the seismic field, they all have certain limitations, especially after earthquake emergencies. The need for earthquake emergency needs with high timeliness requirements, the existing related research results are even more stretched, losing the advantage of massive data, making the role and value of data greatly discounted, and not being able to provide well for emergency decision-making by relevant departments support.

Therefore, artificial intelligence technology is applied to the earthquake emergency network data cleaning, and the artificial intelligence + earthquake emergency network information data cleaning framework is constructed. First of all, according to the disaster assessment report issued by the earthquake emergency expert group, based on the earthquake emergency information corpus and the machine learning accuracy and recall rate evaluation algorithm, construct the data cleaning quality evaluation index; second, the earthquake emergency network Web document dynamically collected by the web crawler technology Data-based, based on HTML structural features, based on TF-IDF Algorithm and Information calculation model, Design the weighted frequency algorithm of earthquake emergency network Web document P-TF-IDF, Filter out single words with low frequency, optimize FastText algorithm N-gram. The feature word vector realizes the rapid text classification of the earthquake emergency network Web document data; third, the data pattern of each attribute of the text classification data is used to construct missing data recognition rules, data classification rules, and data repair rules to detect invalid data in the data set Value, complete data comparison and redundancy judgment, clean up data conflicts and data errors, and generate a complete data set without duplication. The data cleaning framework not only completes the integration of earthquake emergency network information, but also provides a data basis for the visualization of earthquake emergency data, so that massive earthquake data can give full play to its value and significance and provide effective and sufficient data guarantee for decision support to minimize the loss of people's lives and property.

The first section of this paper introduces the background of the project and the advanced concepts and basic structure of the articles of experts in related technical fields.  Section 2 takes data cleaning technology as a clue and introduces how to improve the classification speed of data classification model without losing accuracy.  Section 3 introduces the technical process of data cleaning framework for earthquake emergency network information.  Section 4 introduces the evaluation index of data cleaning quality and P-TF-IDF optimization algorithm and compares and analyses the data cleaning framework of earthquake emergency network information in many ways with the data cleaning rules.  Section 5 puts forward some issues and summaries worth further discussion in the data cleaning framework of the current earthquake emergency network information.

## 2. Data Cleaning Technology

### 2.1. Data Cleaning Technology Based on Artificial Intelligence

Data cleaning is the process of identifying and removing “dirty data” in the data. Take the network data cleaning process as an example, obtain the Web document data set *M* through dynamic web crawler technology, and combine the data quality requirements *R*; after the corresponding data cleaning, find the Web document data set *M*′, *M*′ not only meets all the data quality requirements, but also minimizes the time consumption during the cleaning process and the cleaning cost. In different data application fields, the data quality requirements *R* are used in different forms. In the earthquake emergency network data cleaning requirements *R*′, it is necessary to ensure the timeliness, completeness, and correctness of the data content. Timeliness is mainly reflected in 5 minutes, 10 minutes, half an hour, one hour, 2 hours, and other time nodes after the earthquake occurred. The relevant information of the earthquake event should be continuously summarized; the completeness is mainly reflected in: Web document data. The main data information in the set *M*′ cannot be empty or repeated, and the relevant data meets the constraints of the earthquake emergency data type; the correctness is mainly reflected in: avoiding wrong information in the Web document data set *M*′ during the emergency rescue process to prevent it from causing wrong emergency decision. It is not difficult to see that the core of earthquake emergency needs is to obtain more accurate data in a relatively short period of time to serve emergency decision-making.

The core of the earthquake emergency data cleaning process is the data classification process. Under the requirements of the seismic emergency network data cleaning requirement *R*′, the web document data cleaning is mainly reflected in how to complete the data cleaning in less time and consume the least resources. There are a variety of machine learning classification algorithm models used in the stage, including BP neural network, k-nearest neighbor (KNN), decision tree (Decision Tree), support vector machine, and FastText. BP neural network has a strong advantage in nonlinear mapping, but in order to ensure stability, its learning efficiency is low, and the model training speed is slightly slower than the other algorithms; the k-nearest neighbor (KNN) algorithm has higher accuracy, but its Computational complexity and space complexity are also high, and resource consumption is high; Decision Tree (Decision Tree) Computational complexity lower and better comprehensibility, but excessive phenomena will occur in the matching process; support vector machines are better to implement, but the classification accuracy is poor; FastText supports fast text classification and is compared with the above classification algorithms. To speed up the training speed and test speed, you can use *N*-gram to train the word vector yourself. Since the earthquake emergency network data is mostly Web documents, the earthquake emergency network information data cleaning framework adopts FastText Research on the algorithm model [13–16].

### 2.2. FastText Model

**Figure 1:**
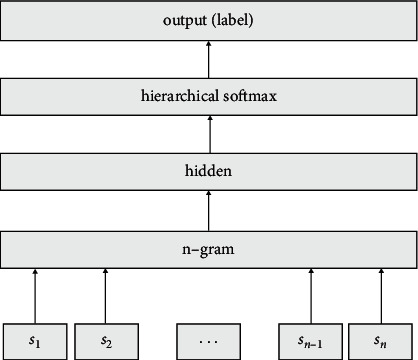
FastText model architecture diagram.

FastText is a machine learning model open source by Facebook in 2016. The model can be applied in the direction of effective text classification of supervised learning, and it can also be applied in the direction of word vector learning of unsupervised learning, and it is mainly used in the direction of text classification. FastText uses Hierarchical SoftMax and *N*-gram to optimize the model. It quickly improves the model training speed and test speed while ensuring high accuracy, and its performance is not inferior to deep learning. The FastText model is mainly divided into three layers, the input layer, the hidden layer, and the output layer. It uses text as the input layer data, and finally the output layer outputs the classification corresponding to the text. The architecture of the FastText model is shown in Figure 1.

The process of FastText model for text classification is as follows: enter the relevant word sequence *S*_1_,*S*_2_,…*S*_*n*−1_, *S*_*n*_ (where *n* is *n* words) of the text to be classified in the input layer, and then use the word vector model *N*-gram (Here, *N* is a multi-element model, commonly used are binary Bi-Gram and ternary Tri-Gram) for the word sequence converted into feature word vectors, and then these feature word vectors are accumulated and averaged, and then mapped to hidden layer, go through Hierarchical SoftMax Perform normalization processing, and finally the FastText model outputs the predicted text classification label. FastText model uses Hierarchical SoftMax and the complexity has been reduced from *N* to log *N*, so how to improve *N*-gram in the execution efficiency of the model is particularly important.

### 2.3. *N*-Gram Model

N-gram is an algorithm based on statistical language models. Among them, *N* is a multi-element model. When *N* = 1, it is a single-element model, when *N* = 2, it is a dual-element model, and when *N* = 3, it is a three-element model, etc. The two-element Bi-Gram model and three Meta's Tri-Gram model are commonly used, such as “The earthquake happened,” when expressed in word granularity, there are only three words in its vocabulary: “the,” “earthquake,” “happened.” At this time, the number of gram represented by the binary Bi-Gram model is 3^2^=9, namely, “The The,” “The earthquake,” “The happened,” “earthquake The,” “earthquake earthquake,” “earthquake happened,” “happened The,” “happened earthquake,” “happened happened.” When *N* in *N*-gram is a specific value, as shown in Table 1.

As shown in Table 1. Gram number statistics table, when the number *V* of the vocabulary is large, there are more feature word vectors that need to be calculated, which can be used in TF-IDF. The algorithm calculates the frequency of words and sorts them, removes some less frequent words, reduces the number of vocabulary *V*, and improves *N*-gram in the execution efficiency of the model.

### 2.4. TF-IDF Algorithm

TF-IDF algorithm is to calculate the word frequency (TF) and the inverse document frequency (IDF the product of) is a statistical method. By counting the frequency of a certain word in a file, it describes the importance of the word in the file and returns the frequency value of the corresponding word. When there are multiple words, the TF-IDF algorithm returns the frequency value of all words. Using TF-IDF, the algorithm calculates the word frequency values of all words in the earthquake emergency network Web document collection, filters out words with smaller word frequency values, and uses important words as the input of the *N*-gram model to perform the feature vector conversion. Although using TF-IDF the algorithm optimizes the N-gram input, which can achieve the speed improvement of the FastText model, but the location factors of keywords such as title, keyword, summary, and hyperlink in the Web document collection of earthquake emergency network are ignored. The location attributes of related words are missing, and the importance of words in the current collection of earthquake emergency network Web documents cannot be fully expressed, resulting in a decrease in the accuracy of label classification. Therefore, this article is based on TF-IDF Algorithm, design the location-weighted frequency algorithm of the feature words of the earthquake emergency network P-TF-IDF. Optimization is performed to ensure that the accuracy is not lost under the premise of improving the classification speed of the FastText model.

## 3. Technical Route

**Figure 2:**
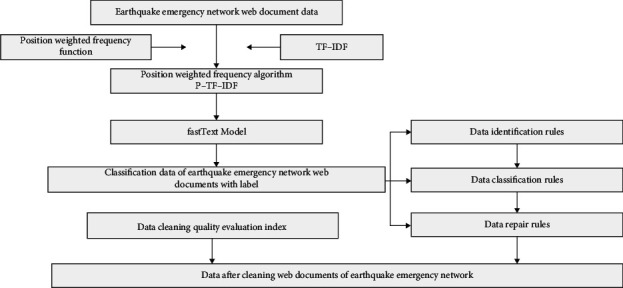
The technical flow chart of the earthquake emergency network information data cleaning framework.

The main process of earthquake emergency network information data cleaning: according to the disaster assessment report issued by the earthquake emergency expert group, based on the earthquake emergency information corpus and the machine learning accuracy and recall rate evaluation algorithm, the data cleaning quality evaluation index is constructed, and the data is dynamically collected based on the web crawler technology. Earthquake emergency network Web document data, refer to HTML structure characteristics, set different positions in the Web document (<title>The title within the label,<meta the abstract described in name = “description” content = “”>, <meta name = “keywords” content = “”>Keywords described in, hyperlinks between <*a*> and </*a*>, paragraph text between <*p*> and </*p*>, etc.) the position weight of related words W, based on the TF-IDF algorithm to improve the word frequency factor Parameters, combined with the information calculation model to improve the location weight *W*, design the location-weighted frequency algorithm P-TF-IDF of the characteristic words of the earthquake emergency network, filter out the words with less word frequency in the earthquake emergency network Web document data, and filter the Web with good word frequency. The document data is used as the input of the FastText model, and the *N*-gram feature word vector in the FastText algorithm is optimized to realize the fast text classification of the seismic emergency network Web document data, and generate the seismic emergency network Web document classification data with Label. After the data recognition rules and data Classification rules, data repair rules, detect invalid values in the data set, complete data comparison and redundancy judgment, clean up data conflicts and data errors, and generate a complete data set without duplication. Finally, the data cleaning quality evaluation index is used to evaluate the quality of the data after the earthquake emergency network Web document cleaning. The data cleaning framework not only completes the integration of the earthquake emergency network information, but also provides a data basis for the visualization of the earthquake emergency data, enabling massive seismic data. It can give full play to its value and significance, and provide effective and sufficient data protection for decision-making support to minimize the loss of people's lives and property. The main process of data cleaning for earthquake emergency network information is shown in Figure 2.

## 4. Seismic Emergency Network Information Data Cleaning Framework

### 4.1. Data Cleaning Quality Evaluation Index

The quality of earthquake emergency information is the key to the success or failure of the earthquake emergency process. Therefore, it is necessary to establish a standardized evaluation standard to evaluate the quality of data cleaning. Based on the disaster evaluation report produced by the earthquake emergency industry as the evaluation index, the data cleaning quality evaluation index is constructed based on the earthquake emergency information corpus and the machine learning accuracy rate and recall rate evaluation algorithm. Cleaning quality mainly refers to the accuracy and comprehensiveness of cleaning. Commonly used evaluation indicators include accuracy, recall, and comprehensive evaluation index *F* value [2], as shown in formula.

(1) $$\begin{array}{c} F=2\times \frac{precision\times recall}{precision+recall}\text{.} \end{array}$$

When using the accuracy rate, recall rate, and comprehensive evaluation index to measure the accuracy of data cleaning, improve the earthquake emergency information corpus, that is, given a database *K* instance with mode *R*, and a database instance that meets all data quality requirements K′. The specific process is as follows:

(1) Based on the disaster assessment report issued by the earthquake emergency expert group, use basic data models such as post-earthquake building damage assessment, damage quantity assessment, natural environment change assessment, secondary disaster investigation, disaster area calculation, rescue resettlement selection and setting *R* establish a statistical table of casualties, building damage, lifeline engineering damage, secondary disaster damage statistics, special engineering damage statistics, and improve the earthquake emergency information corpus *K*.
(2) According to the disaster assessment report issued by the earthquake emergency expert group, fill in the earthquake emergency information corpus *K*, and deliver it to experts in the earthquake emergency field to verify each piece of data, and finally modify it to generate a clean data generation example *K*_*c*_K_*c*_ database.
(3) Based on the earthquake emergency information corpus, use the earthquake emergency information structured data cleaning framework for data cleaning, and fill the cleaned data into the database *K*′ instance. Use K_c_ and *K*′ to calculate the corresponding accuracy rate as formula (2), the recall rate is as shown in formula (3).
  (2) $$\begin{array}{c} precision=\frac{K_{c}\cup K^{\prime }}{K^{\prime }}\text{,} \end{array}$$
  (3) $$\begin{array}{c} recall=\frac{K_{C}\cup K^{\prime }}{K_{C}}\text{.} \end{array}$$

Bring the accuracy rate equation (2) and recall rate equation (3) into equation (1):

(4) $$\begin{array}{c} F=2\times \frac{\left(K_{c}\cup K^{\prime }/\;K^{\prime }\right)\times \left(K_{c}\cup K^{\prime }/\;K_{c}\right)}{\left(K_{c}\cup K^{\prime }/\;K^{\prime }\right)+\left(K_{c}\cup K^{\prime }/\;K_{c}\right)}\text{.} \end{array}$$

After the data cleaning is completed, the data cleaning quality evaluation index is used to evaluate the quality of the data after the cleaning of the earthquake emergency network Web document.

### 4.2. Design P-TF-IDF Algorithm and Optimize FastText Model

Based on the web crawler technology to dynamically collect the seismic emergency network web document data, after preliminary data preprocessing, remove the noise data such as advertisements, refer to the HTML structure features, and structure the title, keywords and abstracts, text, hyperlinks, and other data. The text retains the <*p*> tag. Design the word frequency optimization algorithm P-TF-IDF for location information, including word frequency factor and location factor.

#### 4.2.1. Word Frequency Factor

In the entire Web document, the frequency of words can be divided into high and low. Words with high frequency can better reflect the main purpose of the document. However, the proportion of the number of words in the document to the total number of words cannot fully meet the current needs of Web document analysis. For Web document analysis needs, this article constructs word frequency factor function *tP*_*i*_ based on TF-IDF algorithm as formula:

(5) $$\begin{array}{c} {tP}_{i}=tf_{i,j}*\;\log\;\left(\frac{N_{D1}+N_{D2}+N_{D3}}{n_{i}}\right)\text{,} \end{array}$$

where *tP*_*i*_ is the word frequency factor of the word *t*_*i*_ in the entire document, *tf*_*i*,*j*_ is the word *t*_*i*_ frequency value of the word in the text, *N*_*D*1_ is the number of titles in the entire document, *N*_*D*2_ is the number of natural paragraphs in the entire document (a paragraph between *a* <*p*> and </*p*>), *N*_*D*3_ is the number of hyperlinks in the entire document, and *n*_*i*_ is the sum of the number of titles, natural paragraphs, and hyperlinks that contain words *t*_*i*_ in the text.

#### 4.2.2. Location Factor

In view of the relevant characteristics of the earthquake emergency network Web documents (the positions of some important words in the Web document titles, keywords, abstracts, hyperlinks, etc. are shown in Table 2. Position factor description table), it loses the position attributes of related words. It is unable to fully express the importance of words in the current collection of earthquake emergency network Web documents, resulting in a decrease in the accuracy of label classification.

In order to better calculate the frequency value of words in Web documents and better reflect the words in different positions in Web documents, the following is based on the information calculation model H proposed by Wiener (one of the founders of information theory) as in formula (6) [17], and the structure can be as the information content function *f*_*w*_(*t*_*i*_) describing the word *t*_*i*_ boundary as shown in formula (7), and the realization of the position factor function *tW*_*i*_ is as formula (8).

(6) $$\begin{array}{c} H=P*\;\log_{2}\;M\text{.} \end{array}$$

In formula (6), *H* represents the “information amount;” *P* represents the “probability of the occurrence of this event among all possibilities;” and *M* represents the “total number of all the possibilities of this event.”

(7) $$\begin{array}{c} f_{w}\left(t_{i}\right)=\frac{g_{u}\left(t_{i}\right)*\;\log_{2}\;\left(1+g_{v}\left(t_{i}\right)*l\right)}{\sqrt{\overset{n}{\underset{j=1}{\sum }}\sqrt{g_{u}\left(t_{j}\right)*\;\log_{2}\;\left(1+g_{v}\left(t_{j}\right)*l\right)}}}\left(g_{u}\left(t_{i}\right)\geq 1\;,{\;g}_{v}\left(t_{j}\right)\geq 1\;,l\geq 1\right)\text{.} \end{array}$$

Equation (7) *f*_*w*_(*t*_*i*_) represents the amount of information of the word *t*_*i*_ in different positions; *g*_*u*_(*t*_*i*_) represents the frequency of the word *t*_*i*_ in the Web document,*g*_*v*_(*t*_*i*_) represents the paragraph frequency of the word *t*_*i*_ in the Web document, and l represents the word length of the word *t*_*i*_, where *g*_*u*_(*t*_*i*_), *g*_*v*_(*t*_*i*_), and *l* are both greater than or equal to 1.

(8) $$\begin{array}{c} {tW}_{i}=\alpha +\beta *f_{w}\left(t_{i}\right)\text{.} \end{array}$$

Equation (8) *tW*_*i*_ represents the position factor of the word *t*_*i*_ in different positions, where *α* and *β* represent the information amount coefficient at different positions in the Web document. After a lot of experiments *α* and *β* the coefficient value setting is as shown in Table 3. Web document different position information coefficient *α* and *β* value.

Based on the above-mentioned word frequency factor and location factor, the P-TF-IDF function W(*t*_*i*_) of the word frequency optimization algorithm is constructed as the following equation.

(9) $$\begin{array}{c} W\left(t_{i}\right)={tP}_{i}+{tW}_{i}\text{.} \end{array}$$

Among them, *W*(*t*_*i*_) represents the word frequency value of the word *t*_*i*_ in the Web document, *tP*_*i*_ represents the word frequency factor value of the word *t*_*i*_ in the Web document, and *tW*_*i*_ represents the position factor value of the word *t*_*i*_ in the Web document. Through the function *W*(*t*_*i*_), the word frequency value of all words in the Web document can be calculated and removed. Part of the words with small word frequency values are classified, and the remaining words are classified as the input of the FastText model of the classification model to generate tagged seismic emergency network Web document data, which provides a data basis for further completing the Web data cleaning.

### 4.3. Establish Data Cleaning Rules

After classification based on the FastText model, the tagged seismic emergency network web document data is established to identify missing data, data classification rules, and data repair rules, detect invalid values in the data set, complete data comparison and redundancy judgment, and clean up data conflicts, Data errors, and generate a complete data set without duplication.

#### 4.3.1. Missing Data

Missing data means that some attribute values in the database instance are missing or contain invalid values. Missing data recognition Rule: for invalid values beyond the property field, invalid values in the data set are detected by the data mode of each property. For example, in the statistical table of destruction of secondary disasters in Table 4*T*_4_ [Type] is “N/A.” It is worth noting that *T*_2_ [Range of influence] is also a missing value because “Null” is not a valid expression of the scope of influence. The detection of missing data is relatively simple. It only needs to check whether the attribute value that is not allowed to be empty is empty, “Null” Or “N/A.”

#### 4.3.2. Data Redundancy

Data redundancy means that the same data appears multiple times in a database instance, that is, there is duplication between data. Establish data classification rules: group according to tags, query redundant data in the same group, and select the last record according to the data release time. For example, tuples *T*_1_ and *T*_3_ in the statistical table of secondary disaster damage *T*_3_. They all represent the secondary disaster damage information of Jiuzhaigou Scenic Area, Sichuan, and the last record of the time is selected for preservation.

#### 4.3.3. Data Conflict and Data Error

There is a data conflict between the two pieces of data that cannot satisfy the integrity constraint. Establish repair rules, as shown in formula (10), where: *X* is a subset of attribute set *Q*, *t*_*p*_[*X*] is a possible value of attribute set *X*, representing the evidence value; attribute *Y* belongs to *Q*\*Y*, *t*_*p*_^−^[*Y*]is a set of constants in the domain of *Y*, representing the wrong value of *Y*, and *t*_*p*_^+^[*Y*] belongs to the domain of *Y*, but does not belong to *t*_*p*_^−^[*Y*], *t*_*p*_^+^[*Y*] means the correct value of *Y*.

Take Table 4 damage statistics of secondary disasters as an example, *Q* = {“Place name,” “Types of,” “Destruction,” “Sphere of influence”}, *X* = {“Place name”}, *Y* = {“Types of,” “Destruction,” “Sphere of influence”}; Settings *t*_*p*_[*X*] = {“Jiuzhaigou Scenic Area, Sichuan”}, t_p_^−^[*Y*] = {(“landslide,” “severe,” “null”), (“landslide,” “null,” “null”), (“Barrier lake,” “null,” “null”)…}, t_p_^+^[Y] = {“Barrier lake,” “severe,” “Jiuzhaigou Scenic Area”}, When the data tuple *t* = *T*_2_ when using repair rules for data cleaning, *t*[*X*] is equal to *T*_*p*_[*X*], *t*[*Y*]*εt*_*p*_^−^[*Y*], *T*_2_ is the noise data, delete the noise data *T*_2 ._

(10) $$\begin{array}{c} \phi =\left(\left(X,t_{p}\left[X\right]\right),\left(Y,t_{p}^{-}\left[Y\right]\right)\right)⟶t_{p}^{+}\left[Y\right]\text{.} \end{array}$$

After classification based on the FastText model, generate tagged seismic emergency network web document data, data identification rules, data classification rules, and data repair rules, and perform data cleaning to generate a complete data set without duplication.

### 4.4. Cleaning Results and Analysis

#### 4.4.1. Data Preprocessing

To better verify the cleaning effect, select “Yushu Earthquake,” “Tibet Earthquake,” “Wenchuan Earthquake,” “Jiuzhaigou Earthquake,” “Ludian Earthquake”, and other major earthquakes after the earthquake industry dedicated network information, news media, etc. Website 13,644 Web data such as HTML, used for FastText model training and test, go through data preprocessing.Pretreatment data as is shown in Table 5.

#### 4.4.2. P-TF-IDF Algorithm Optimization FastText Model

**Figure 3:**
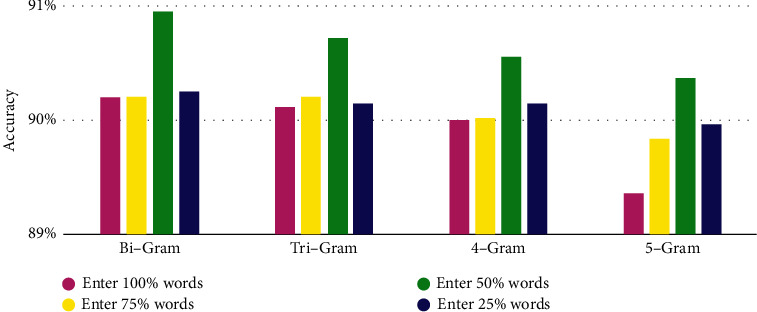
Comparison of accuracy of inputting different numbers of words after filtering according to weight.

**Figure 4:**
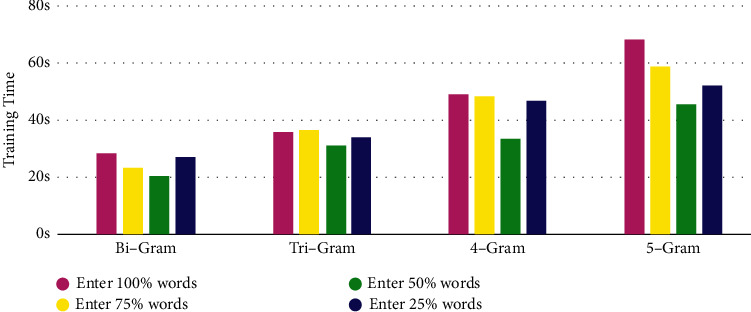
Comparison of training time of model with different number of words input after weight filtering.

For 13644 web data such as HTML after preprocessing, use P-TF-IDF's word frequency factor and location factor, compute network Web Data per word of Weights data value, and filter according to the weight data value lose partial word frequency and location nun important of Words, and then perform model training, and the training results are shown inTable 6. The comparison of the accuracy rate of different numbers of words input after filtering according to the weight is shown in Figure 3, the comparison of the training time of different numbers of words input after filtering according to the weight is shown in Figure 4.

#### 4.4.3. Data Cleaning

After optimization using P-TF-IDF algorithm, FastText model data is classified and carried out in accordance with the rules of the data cleaning framework, using “Yun nan Da li Yang bi earthquake” 15871 strip data and “Qing hai Guo luo zhou Ma duo earthquake” 7961 strip data conduct cleaning, combined data cleaning quality evaluation index *F* conduct a comprehensive evaluation, conducted an evaluation and comparison, the results are shown in Table 7.

The experimental results show that through the P-TF-IDF algorithm optimization FastText model to take the Bi-Gram model as an example, when filtered out 50% Words (input 50%, word quantity for 91711). Compared with the case where word frequency factor and position factor are not added (input 100%, number of words is 191297), the time is 7.57 seconds faster and the accuracy rate is increased by 0.76%; compared with the case where 25% words are filtered (input 75%, number of words is 132660), the time is 2.83 seconds faster and the accuracy rate is increased by 0.76%; compared with the case where 75% words are filtered (input 25%, number of words is 47071), the time is 6.83 seconds faster and the accuracy rate is increased by 0.71%. This article chooses Add to Word frequency factor and location factor, and 50% of words are reserved for model training, which guarantees a high accuracy rate under the premise of effectively improving the model training time. Using the earthquake emergency network information data cleaning framework, 15871 pieces of data of “Yun nan Da li Yang bi earthquake” and 7961 pieces of data of “Qing hai Guo luo zhou Ma duo earthquake” are cleaned. From the results, it is not difficult to see that only the word frequency factor is used to optimize the model. Compared with the traditional FastText model for data classification, the time of “Yun nan Da li Yang bi earthquake” is shortened by 1.98 seconds. The comprehensive evaluation index increased by 13.08%, the time of “Qing hai Guo luo zhou Ma duo earthquake” was shortened by 1.94 seconds, and the comprehensive evaluation index increased by 15.41%. After adding the location factor, although the time increased slightly, the comprehensive evaluation index of “Yun nan Da li Yang bi earthquake” increased by 18.21%, and the comprehensive evaluation index of “Qing hai Guo luo zhou Ma duo earthquake” increased by 19.06%. In the location factor, different locations have different impacts on the comprehensive evaluation indicators of the data cleaning framework. Titles, hyperlinks, and paragraph text have a greater impact. It is not difficult to see that some websites have abstracts and keywords described in HTML. But inconsistent with the theme of the Web document, the abstract and keywords have little effect on the accuracy of cleaning.

## 5. Conclusion

In the context of the advent of the big data era and the development of emerging technologies, the integration of artificial intelligence technology and earthquake emergency network information ensures the comprehensiveness and integrity of the data, so that the earthquake emergency work can be comprehensive and accurate in the shortest time, timely disaster information, make scientific and reasonable decisions and put them into action. This article is based on Web document data of earthquake emergency network dynamically collected by web crawler technology, and use TF-IDF Algorithm design word frequency factor, use information calculation model to calculate the amount of information of each word, describe the boundary of each word through the amount of information and design location factor, combining word frequency factor and location factor, and designed a Location Weighted Frequency Algorithm P-TF-IDF in Web Documents, P-TF-IDF reduces the number of words in the input layer of n-gram in FastText model and improves the classification efficiency of FastText model and outputs labeled classification data, using data identification rules, data classification rules, and data repair rules to complete data cleaning. Under the premise of ensuring the cleaning effect of the earthquake emergency network information data cleaning framework, the calculation time is shortened. The cleaning framework is suitable for loosely structured Web document page data and other text data with standardized formats and has certain practicability. Currently, the data cleaning framework is only a preliminary cleaning of the earthquake emergency network web documents, and the cleaning results need to be verified multiple times to check the correctness of the algorithm. Therefore, further research is needed.

## Figures and Tables

**Table 1:** Gram number statistics table.

*N*	Vocabulary list size *V* (*V* = 40,000 words)	Formula *V*^*N*^	Number of gram
*N* = 2 (Bi-gram)	40000	40000^2^	**1,600,000,000**
*N* = 3 (Tri-gram)	40000	40000^3^	6.4 ∗ 10^13^
*N* = 4 (4-gram)	40000	40000^4^	2.56 ∗ 10^18^

**Table 2:** Position factor description table.

Location	HTML document location	Description of the amount of relevant location information
Title	<title></title> tag	The core idea and theme of the document
Abstract	<meta name = “description” content = “”>Attribute value	The abstract is a summary of the main purpose of the document
Key words	<meta name = “keywords” content = “”>attribute value	Keywords are nominal terms that reflect the main content of the full text
Hyperlink	Text between <*a*>and</*a*>	A hyperlink is a web link title that is similar to the subject of the document
Paragraph	Text between <*p*>and</*p*>	Document body

**Table 3:** Information coefficient *α* and *β* value table for different positions of web documents.

Location	*α* value	*β* value
Title	1	0.6
Abstract, keywords	0	1.6
Hyperlink	0	1.7
Paragraph	0	1.2

**Table 4:** The statistical table of damage caused by secondary disasters.

Tuple	Place name	Types of	Destruction situation	Sphere of influence
*T *_1_	Jiuzhaigou scenic area, Sichuan	Barrier lake	Severe	Jiuzhaigou scenic area
*T *_2_	Jiuzhaigou scenic area, Sichuan	Landslide	Severe	Null
*T *_3_	Jiuzhaigou scenic area, Sichuan	Barrier lake	None	The blocked river has flowed normally
*T *_4_	Sichuan Jiuzhaigou county	N/A	Severe	Heihe, Shuangle, Anle, and other towns
*T *_5_	Jiuzhaigou scenic area, Sichuan	Mudslide	Severe	Jiuzhaigou scenic area panda sea

**Table 5:** Pretreatment data.

HTML page	*H*1 label (title)	A label (hyperlink)	Abstract, keywords	*P* label (paragraph)	Words
**13644**	**13122**	**769222**	**51277**	**206683**	**191297**

**Table 6:** Model training result efficiency compared.

*N*-gram model (N)	Number of gram	Feature factor selection in P-TF-IDF algorithm	Accuracy	Training time (s)	P-TF-IDF after filtering enter percentage of words (%)	Word count
*N* = 2 (Bi-gram)	2.2 ∗ 10^9^	Add word frequency factor and location factor	0.9026	27.52	25	47071
*N* = 3 (Tri -gram)	1.0 ∗ 10^14^	Add word frequency factor and location factor	0.9015	34.47		
*N* = 4 (4-gram)	4.9 ∗ 10^18^	Add word frequency factor and location factor	0.9015	47.17		
*N* = 5 (5-gram)	2.3 ∗ 10^23^	Add word frequency factor and location factor	0.8997	52.55		

*N* = 2 (Bi-gram)	**8.4 ∗ **10^9^	Add word frequency factor and location factor	**0.9097**	**20.69**	**50**	**91711**
*N* = 3 (Tri -gram)	**7.7 ∗ **10^14^	Add word frequency factor and location factor	**0.9073**	**31.22**		
*N* = 4 (4-gram)	**7.0 ∗ **10^19^	Add word frequency factor and location factor	**0.9056**	**33.64**		
*N* = 5 (5-gram)	**6.4 ∗ **10^24^	Add word frequency factor and location factor	**0.9038**	**45.79**		

*N* = 2 (Bi-gram)	1.7 ∗ 10^15^	Add word frequency factor and location factor	0.9021	23.52	75	132660
*N* = 3 (Tri -gram)	2.3 ∗ 10^15^	Add word frequency factor and location factor	0.9021	36.67		
*N* = 4 (4-gram)	3.0 ∗ 10^20^	Add word frequency factor and location factor	0.9003	48.61		
*N* = 5 (5-gram)	4.1 ∗ 10^25^	Add word frequency factor and location factor	0.8985	58.81		

*N* = 2 (Bi-gram)	3.6 ∗ 10^10^	None	0.9021	28.26	100	191297
*N* = 3 (Tri -gram)	7.0 ∗ 10^15^	None	0.9011	36.31		
*N* = 4 (4-gram)	1.3 ∗ 10^21^	None	0.9001	49.59		
*N* = 5 (5-gram)	2.5 ∗ 10^26^	None	0.8937	68.58		

**Table 7:** Data cleaning comprehensive evaluation result.

Feature factor selection in P-TF-IDF algorithm	Comprehensive evaluation index *F* value		Data cleaning time (s) (excluding data preprocessing time)	
	Yun nan Da li Yang bi earthquake	Qing hai Guo luo zhou Ma duo earthquake	Yun nan Da li Yang bi earthquake	Qing hai Guo luo zhou Ma duo earthquake
Add word frequency factor	84.68%	85.31%	2.39	1.27
Add word frequency factor and location factor	**89.81%**	**88.96%**	**2.52**	**1.78**
Remove the hyperlink factor part	78.87%	79.03%	2.61	1.69
Remove the title factor part	77.32%	76.22%	2.58	1.91
Remove the keyword factor part of the summary	82.40%	83.80%	2.46	1.43
Remove the paragraph factor part	74.70%	75.30%	2.41	1.32
Traditional FastText model	71.60%	69.90%	4.37	3.21

## Data Availability

No data were used to support this study.
